# New insights into bacterial mechanisms and potential intestinal epithelial cell therapeutic targets of inflammatory bowel disease

**DOI:** 10.3389/fmicb.2022.1065608

**Published:** 2022-12-16

**Authors:** Bing Liang, Changhao Wu, Chao Wang, Wenshe Sun, Wujun Chen, Xiaokun Hu, Ning Liu, Dongming Xing

**Affiliations:** ^1^Cancer Institute, The Affiliated Hospital of Qingdao University, Qingdao, China; ^2^Department of Biochemistry and Physiology, Faculty of Health and Medical Sciences, University of Surrey, Guildford, United Kingdom; ^3^Intervention Neurosurgery, The Affiliated Hospital of Qingdao University, Qingdao, China; ^4^School of Life Sciences, Tsinghua University, Beijing, China

**Keywords:** inflammatory bowel disease, gut microbiota, bacterial mechanisms, intestinal epithelial cells (IECs), therapeutic targets

## Abstract

The global incidence of inflammatory bowel disease (IBD) has increased rapidly in recent years, but its exact etiology remains unclear. In the past decade, IBD has been reported to be associated with dysbiosis of gut microbiota. Although not yet proven to be a cause or consequence of IBD, the common hypothesis is that at least some alterations in the microbiome are protective or pathogenic. Furthermore, intestinal epithelial cells (IECs) serve as a protective physical barrier for gut microbiota, essential for maintaining intestinal homeostasis and actively contributes to the mucosal immune system. Thus, dysregulation within the intestinal epithelium increases intestinal permeability, promotes the entry of bacteria, toxins, and macromolecules, and disrupts intestinal immune homeostasis, all of which are associated with the clinical course of IBD. This article presents a selective overview of recent studies on bacterial mechanisms that may be protective or promotive of IBD in biological models. Moreover, we summarize and discuss the recent discovery of key modulators and signaling pathways in the IECs that could serve as potential IBD therapeutic targets. Understanding the role of the IECs in the pathogenesis of IBD may help improve the understanding of the inflammatory process and the identification of potential therapeutic targets to help ameliorate this increasingly common disease.

## Introduction

Inflammatory bowel disease (IBD) is a multifactorial chronic inflammatory disease, which primarily includes Crohn’s disease (CD) and Ulcerative colitis (UC). IBD causes severe gastrointestinal (GI) tract symptoms, including diarrhea, abdominal pain, bleeding, anemia, and weight loss, and poses a severe but incurable threat to human life and health. The etiology and pathogenesis of IBD remain unknown. However, they may be linked to genetic susceptibility, intestinal microbial homeostasis imbalance, impaired intestinal mucosal barrier function, disorders of innate and adaptive immune regulation in the intestine, and stimulation by external environmental factors. IBD primarily affects genetically predisposed individuals in a specific environment where the intestinal mucosal immune system produces an abnormally amplified immune response to intestinal microbial antigens, resulting in inflammatory damage to the intestinal mucosa.

There are ~400 species of bacteria in the human intestine, including resident and some transient bacteria. The sites of IBD are the colon, rectum, and ileum, where the intestine is most exposed to bacteria. Some clinical features of IBD are similar to those of some infectious diseases of the GI. Several studies have shown that a large quantity of bacteria in the human intestine affects the function of the intestinal mucosal immune system and that a large number of intestinal antigens, such as enteric bacteria and bacterial products, induce abnormal immune responses in the intestine of individuals with genetic susceptibility to IBD, leading to the development of IBD. Although the current research data do not support that infection with a specific pathogenic microorganism causes the development of IBD, clinical observations suggest that intestinal infections caused by these microorganisms can induce the recurrence of IBD in remission. In addition, many experiments have confirmed the involvement of intestinal bacteria in the development of experimental colitis. About 99.9% of human GI microbiota are bacteria, and the other 0.1% are fungi, archaea, and viruses (Qin et al., 2010; Sommer and Backhed, 2013). Bacteria have been extensively reported to have an important role in the pathogenesis of IBD. Intestinal epithelial cells (IECs) line the surface of the intestinal epithelium and perform a variety of functions, including the physical isolation of commensal bacteria and the integration of microbial signals (Peterson and Artis, 2014). IECs also react to immune cell-produced factors that regulate epithelial barrier function, proliferation, and differentiation (Soderholm and Pedicord, 2019). Dysregulation of the IECs can lead to increased intestinal permeability, abnormal IEC interactions with immune cells, and disruption of intestinal immune homeostasis, all of which are associated with the clinical course of IBD (Coskun, 2014).

Many medications, including aminosalicylates, corticosteroids, immunomodulatory drugs, biologics, and antibiotics, can help reduce inflammation and alleviate IBD symptoms. Among them, the introduction of tumor necrosis factor (TNF) inhibitors is an outstanding achievement, allowing for long-term remission and modification of the IBD course in many patients (Nielsen and Ainsworth, 2013). The clinical treatment of IBD has advanced dramatically due to the development and application of these medications and the updating of therapeutic targets. However, these conventional treatments still focus on inducing and maintaining clinical remission, which is not yet wholly curable. A substantial proportion of patients still have long-term chronic active inflammatory reactions and the need for surgical procedures, which significantly impacts patients’ quality of life and imposes a major social and medical burden. Therefore, it is critical to investigate the pathogenesis of IBD, identify new targets for IBD treatment and implement precision medicine to develop the best treatment for patients.

## Bacteria species and functional pathways involved in the protection and pathogenicity of inflammatory bowel disease

The gut microbiota is considered to be a nonnegligible factor in the pathogenesis of IBD and an important target for the research of therapeutic drugs for IBD, which can regulate the host’s vital activities, modulate the immune response and counteract the dysbiosis of gut microbiota, and play a vital role in protecting the host’s health. Many studies have found that IBD patients have a disturbed intestinal microecological balance, characterized by reduced diversity, increased bacterial instability, increased *Actinobacteria,* and *Proteobacteria*, and decreased *Bacteroidetes* and *Firmicutes*, especially a significant decrease in bacteria producing short-chain fatty acids (SCFAs; Caruso et al., 2020). The pathophysiological mechanisms by which gut microbiota antigens induce abnormal responses in the innate and adaptive immune systems of the intestinal mucosa remain unknown. In addition to SCFAs, tryptophan metabolism, bile acid metabolism, Polysaccharide A (PSA) production, and virulence factor genes are balanced in the normal intestine, whereas tryptophan derivatives are reduced, secondary bile acids are reduced, *PSA* gene expression is reduced, and microbial virulence gene expression is increased in IBD patients’ intestines.

### Bacteria species and functional pathways that are potentially protective of inflammatory bowel disease

**Figure 1:**
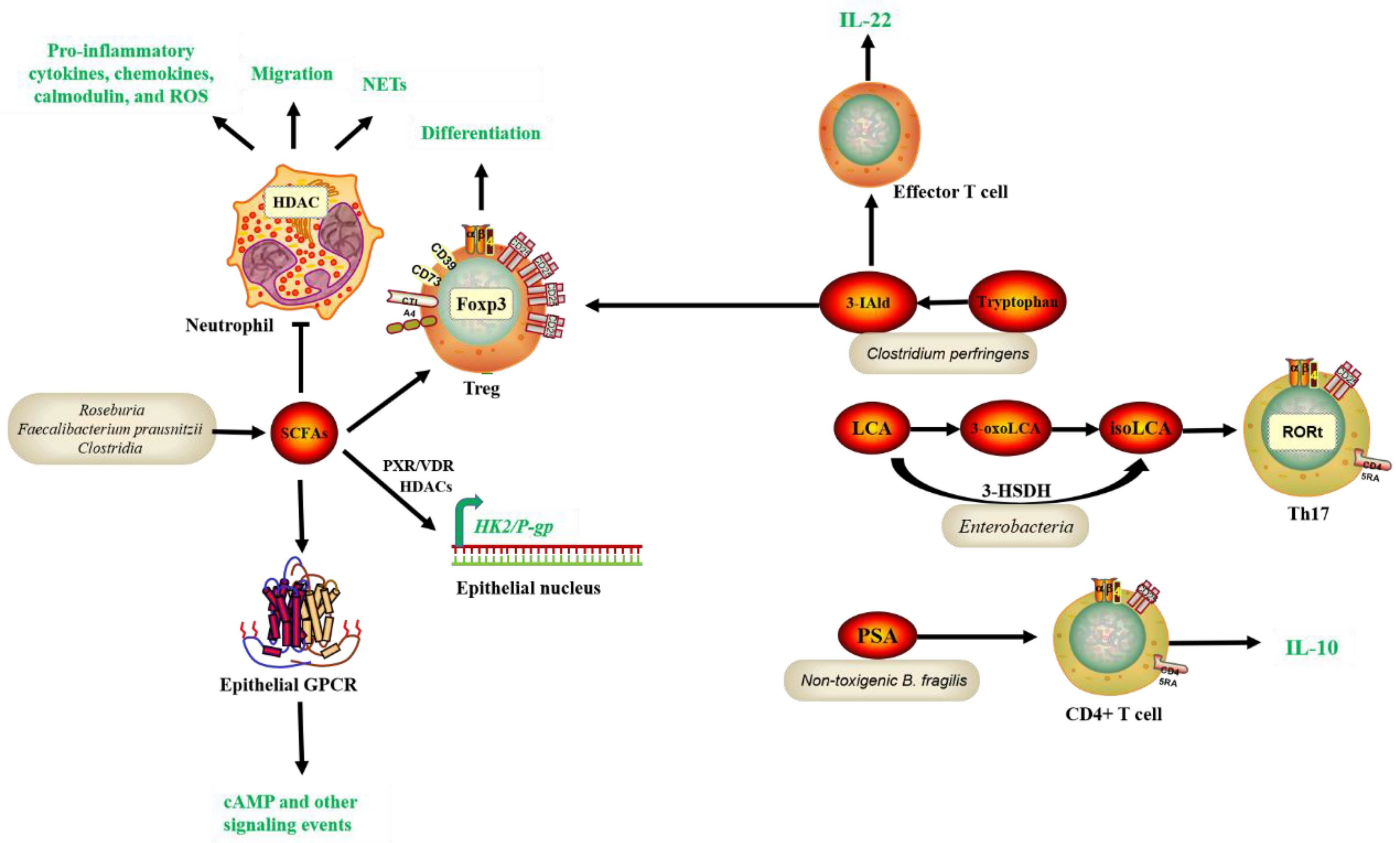
Common bacteria species and functional pathways involved in the protection of IBD. SCFAs, tryptophan, and bile acid metabolites, and PSA production by gut microbiota interact with receptors on IECs and immune cells, contributing to gene regulation, epithelial function, and homeostasis maintenance and inflammation modulation, thus leading to alleviation of IBD symptoms. Short-chain fatty acids (SCFAs), Polysaccharide A (PSA), Histone deacetylase (HDAC), Foxhead box P3 (Foxp3), Neutrophil extracellular traps (NETs), Hexokinase 2 (HK2), P-glycoprotein (P-gp), Intestinal epithelial cells (IECs), Pregnane X receptor (PXR), Vitamin D3 receptor (VDR), Helper T-cell 17 (Th17), 3-hydroxysteroid dehydrogenase (3-HSDH), Retinoid-related orphan receptor gamma t (RORt), indole-3-carboxaldehyde (3-IAld), Aryl hydrocarbon receptor (Ahr).

The first bacterial metabolite to play a potentially protective role for IBD is SCFA. The major SCFAs, including acetate, propionate, and butyrate, account for over 95% of the total SCFA content in feces (Kim et al., 2019). SCFAs can be produced by bacteria such as *Roseburia* and *Faecalibacterium prausnitzii*. Intestinal microorganisms convert dietary fiber fatty acids into SCFAs, which then enter the intestinal lumen. SCFAs can bind to G protein-coupled receptors (GPCRs), like GPR41 (Brown et al., 2003), GPR43 (Maslowski et al., 2009), and GPR109A (Thangaraju et al., 2009), to activate signaling cascades that control immune functions and improving intestinal barrier integrity (Vieira et al., 2015). SCFAs can also modulate immune cells, reduce pro-inflammatory factors, and mitigate the development of IBD (Luis et al., 2021); (Renga et al., 2022); (Rösch et al., 2017); (Nicolas and Chang, 2019). Back in 2013, butyrate, a by-product from *Clostridia* fermentation of indigestive dietary fiber, was discovered to increase histone H3 acetylation at the *Foxhead box P3 (Foxp3)* promoter and conserved noncoding regions, promoting Treg differentiation and alleviate the development of colitis in mice (Furusawa et al., 2013). Similarly, Li et al. reported that butyrate inhibited neutrophil migration, formation of neutrophil extracellular traps (NETs), and production of pro-inflammatory cytokines, chemokines, and calmodulin in IBD patients by inhibiting histone deacetylase (HDAC). RNA sequencing analysis revealed that butyrate’s immunomodulatory effects on neutrophils included leukocyte activation, innate immune response regulation, and oxidative stress (Li et al., 2021). Recent studies demonstrated previously unknown intracellular signaling network mediated by butyrate. For instance, butyrate could exert a protective effect against colitis by downregulating Hexokinase 2 (HK2) in the intestinal epithelium *via* HDAC8 (Hinrichsen et al., 2021). Moreover, a subsequent study revealed that butyrate increased *P-glycoprotein (P-gp)* transcription by inhibiting HDACs and activating nuclear receptors, pregnane X receptor (PXR) and vitamin D3 receptor (VDR). P-gp is a component of Intestinal epithelial cells (IECs) that plays an essential role in the excretion of endogenous cannabinoid toxins, the prevention of excessive inflammation, and the maintenance of endocytosis (Foley et al., 2022; Figure 1). The second pathway that protects against IBD is the metabolism of bile acids. Bile acids are synthesized in the liver, then processed and modified before being secreted into the duodenum, where intestinal microorganisms convert them to secondary bile acids (Ahmad and Haeusler, 2019). There are two secondary bile acids: lithocholic acid (LCA) and chenodeoxycholic acid (CDCA). They modulate immune cells, reduce pro-inflammatory factors, decrease systemic inflammation and alleviate the development of IBD (Postler and Ghosh, 2017); (Sinha et al., 2020). Bile acids can affect host metabolism, cancer progression, and innate immunity, but their effects on adaptive immune cells such as Helper T-cell 17 (Th17) cells and Regulatory T (Treg) cells have not been clarified. Previous research has shown that 3-oxoLCA and isoalloLCA, 2 lithocholic acid (LCA) derivatives, can inhibit the differentiation of Th17 cells and promote the differentiation of Treg, respectively. 3-oxoLCA inhibits the differentiation of Th17 cells by directly binding to the key transcription factor RAR-related orphan receptor gamma t (RORγt) and isoalloLCA promotes Treg differentiation by enhancing *Foxp3* expression and inducing the production of mitochondrial reactive oxygen species (mROS; Hang et al., 2019); however, the relationship between 3-oxoLCA and IBD remains unknown. A recent study discovered *Enterobacteria* and bacterial enzyme 3-hydroxysteroid dehydrogenase (3-HSDH) that convert LCA to 3-oxoLCA and then to isoLCA through *in vitro* culture screening. IsoLCA, like 3-oxoLCA, inhibits the transcriptional activity of *RORγt* and thus reduces Th17 cell differentiation (Paik et al., 2022). This study adds to our understanding of *Enterobacteria*-metabolite-host immune interactions and highlight the critical role of microbe-derived bile acid metabolites in regulating host immunity, shedding new light on the treatment of immune diseases such as IBD (Figure 1). Treg cells have an important role in maintaining intestinal homeostasis, and their differentiation is influenced by the products of gut microbiota. A recently published study identified two new secondary bile acids, ω-muricholic acid (ω-MCA) and 3β-hydroxydeoxycholic acid (isoDCA) that effectively induce Treg differentiation *in vitro*, with isoDCA being more abundant in the intestine (Campbell et al., 2020). Further studies showed that isoDCA enhances its Treg induction by interacting with the farnesol X receptor (FXR) in dendritic cells (DCs), and by constructing engineered bacteria and colony colonization experiments, it was confirmed in mice that isoDCA-producing colonies promote peripheral Treg. The third pathway is tryptophan metabolism. Tryptophan is a necessary amino acid. Dietary tryptophan can undergo the kynurenine ammonia pathway in response to microbial and other inflammatory stimuli, producing a variety of compounds (Ma et al., 2020). Microbial action breaks down tryptophan to an indole derivative, which can modulate immune cells by stimulating IL-22, secreting antimicrobial peptides, and inhibiting the expression of inflammatory factors (Zelante et al., 2013). *Clostridium perfringens* can participate in the metabolism of tryptophan, which is also protective of IBD. According to a recent study, a tryptophan metabolite, indole-3-carboxaldehyde (3-IAld) reduces immune checkpoint inhibitor (ICI)-induced colitis in mice by influencing the structure and function of the mouse gut microbiota, resulting in an increase in sugar-fermenting bacteria and SCFA-producing bacteria and regulating Aryl hydrocarbon receptor (Ahr)/IL-22, and IL-10+ Treg cells (Renga et al., 2022; Figure 1). PSA production, the fourth potentially protective pathway for IBD, is primarily produced by *Bacteroides fragilis*. *B. fragilis* can deliver the immunomodulatory molecule PSA to immune cells by releasing outer membrane vesicles (OMVs), activate TLR 2 on the surface of DCs, resulting in the release of cytokines that promote IL-10 production by T cells (Wang et al., 2006; Shen et al., 2012; Dasgupta et al., 2014). This process is enhanced by direct binding of PSA to TLR2 expressed on Foxp3+ Tregs, which increases IL-10 production even further (Round and Mazmanian, 2010; Figure 1). PSA-induced IL-10 production inhibits the activity of mucosal effector T cells, particularly TH17 cells, and thus protects mice in colitis models (Mazmanian et al., 2008; Dasgupta et al., 2014). Here, *B. fragilis* refers mainly to non-toxigenic *B. fragilis*, or NTBF. On the other hand, Enterotoxigenic B. fragilis (ETBF) is toxin-producing, which is likely to promote the development of IBD (Chung et al., 2018).

**Figure 2:**
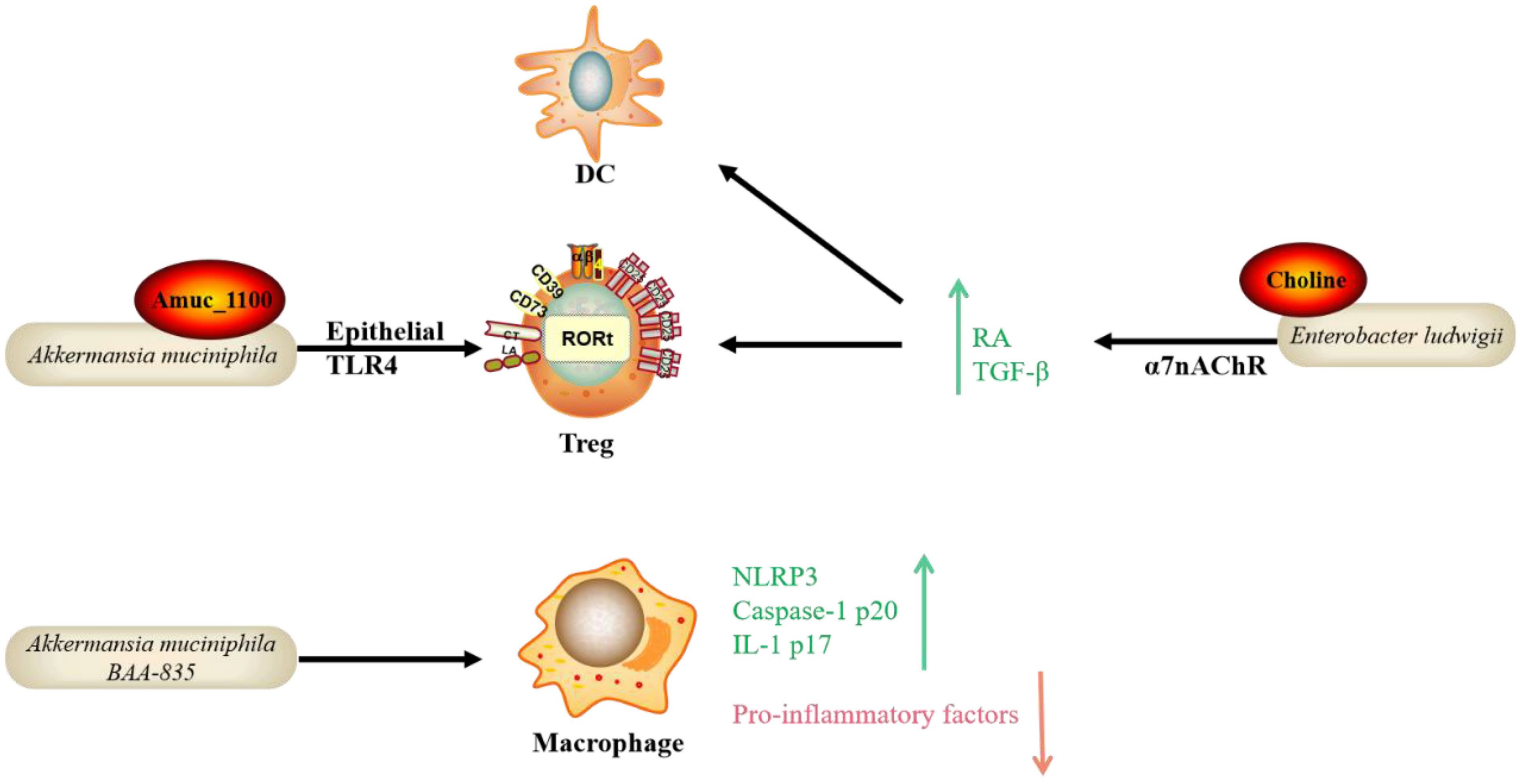
Emerging bacteria species and functional pathways involved in the protection of IBD. In addition to the well-known mechanisms, emerging bacteria species and functional molecules/pathways identified recently possess the potential to protect hosts from IBD. The mode of action includes the bacterial own component interacting with receptors on IECs and immune cells with the participation of Treg, DC and macrophage. Toll-like receptor 4 (TLR4), Retinoic acid (RA), NLR family pyrin domain containing 3 (NLRP3), Dendritic cell (DC), α7 nicotinic acetylcholine receptor (α7nAChR).

In addition to the four metabolite pathways associated with gut microbiota mentioned previously, other microbial species and functional molecules/pathways have been studied extensively recently (Figure 2). *Akkermansia muciniphila* (Akk) binds to a receptor on the membrane of IECs, affecting downstream immune regulatory responses, lowering lipopolysaccharide (LPS), reducing inflammation, regulating lipids, and lowering blood glucose in mouse models (Plovier et al., 2017). The literature has reported that Akk and its outer membrane protein, Amuc_1100, can alleviate enteritis in mice (Zhai et al., 2019). Toll-like receptor 4 (TLR4) is a critical mediator in the interactions between gut microbiota and host immunity. Yang et al. recently discovered that TLR4 protects mice from colitis by promoting Akk colonization in the intestine, which upregulates RORt+ Treg cell-mediated immune responses (Liu et al., 2022). Furthermore, in a previous study, oral administration of Akk bacteria strain BAA-835 significantly improved the phenotype of Dextran sulfate sodium (DSS)-induced acute colitis in mice. Mechanistically, Akk increased the expression of *NLR family pyrin domain containing 3 (NLRP3), caspase-1 p20*, and *IL-1 p17* improved intestinal barrier function, and inhibited the expression of pro-inflammatory factors such as *TNF-α* and *IL-6* (Qu et al., 2021). However, an earlier study found that Akk can cause colitis in sterile IL-10 gene-deficient mice (Seregin et al., 2017). Therefore, whether Akk can be used to treat IBD is still debatable. Furthermore, according to a recent study by Wang et al., *Enterobacter ludwigii* isolated from metronidazole-treated mouse feces could enhance DCs and promote Tregs differentiation through its metabolite choline and its receptor α7 nicotinic acetylcholine receptor (α7nAChR)-mediated upregulation of retinoic acid (RA) and TGF-β, thereby increasing the CD103+ DC/Treg-dependent tolerance response and ultimately reducing the susceptibility of mice to DSS-induced colitis. Thus, this study provides a potential therapeutic approach for IBD (Li et al., 2022).

### Bacteria species and functional pathways that are potentially promotive of inflammatory bowel disease

**Figure 3:**
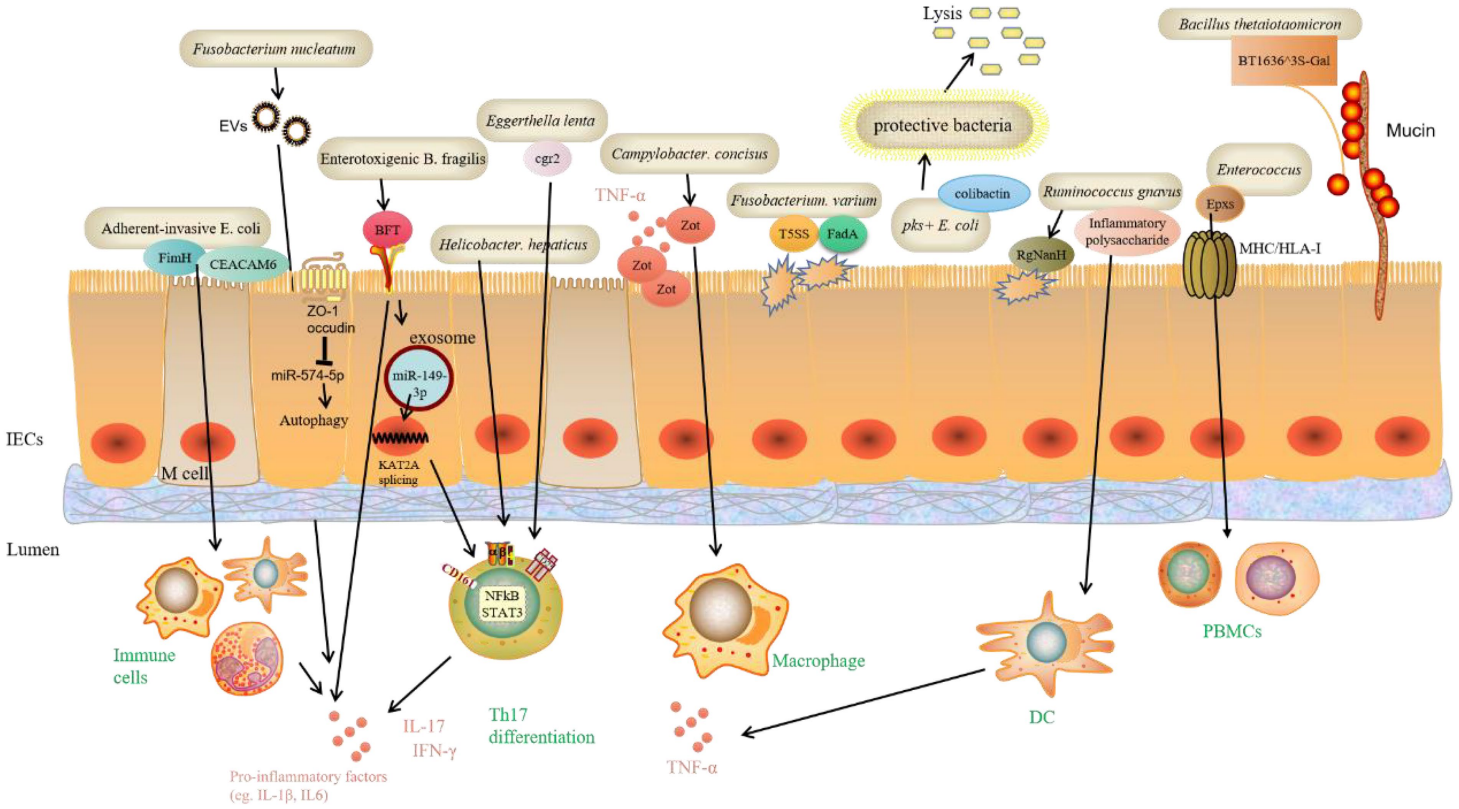
Bacteria species and functional pathways that are potentially promotive of IBD. Recent research identified many new bacteria species and the virulence factors they contain that potentially promote IBD. These virulence factors can directly bind to receptors on IECs and damage barrier function. Specific miRNAs are involved in regulating signaling pathways that further regulate immune cells to alter normal inflammatory responses. Moreover, bacteria-expressed sulfate esterases catalyze mucin O-glycans, which leads to mucin degradation, and ultimately worsens IBD. Carcinoembryonic antigen-related cell adhesion molecule 6 (CEACAM6), *Bacteroides fragilis* toxin (BFT), PHD finger protein 5A (PHF5A), Histone acetyltransferase KAT2A, Type 5 secretion systems (T5SS), FadA adhesion gene, Pore-forming toxins (Epxs), Human leukocyte antigen class I (HLA-I), Peripheral blood mononuclear cells (PBMCs), Intramolecular trans-sialidase (RgNanH), Sulfate esterase (BT1636^3S-Gal), tight junction proteins (ZO-1 and occludin), Cardiac glycoside reductase (cgr2).

Previous research has found that some pathobionts play a pro-IBD role in genetically susceptible individuals, such as *Mycobacterium paratuberculosis* and *Helicobacter pylori*. In addition, the efficacy of antibiotics and probiotics targeting gut microbiota further suggests a role for microorganisms in IBD. New research has recently emerged identifying many new bacteria species and the virulence factors they contain that potentially promote IBD (Figure 3). Adherent-invasive *E. coli* (AIEC) is distinct from typical *E. coli*, which AIEC is enriched in the intestinal microbiota of CD patients and promotes intestinal inflammation, development of IBD and even colorectal cancer (CRC). However, it is unclear how the nutrient metabolism of AIEC affects intestinal homeostasis. AIEC can adhere to the IECs layer *via* the FimH and carcinoembryonic antigen-related cell adhesion molecule 6 (CEACAM6), enter the lamina propria and Peyer’s patch *via* Microfold (M) cells, and interact with immune cells in the intestinal lumen to increase the production of inflammatory factors (Barnich et al., 2007; Palmela et al., 2018; Viladomiu et al., 2021). A recent article reported that in CD, propionate metabolism by AIEC is critical in promoting intestinal T cell inflammation. Meanwhile, CX3CR1+ mononuclear phagocytes produce IL-1β upon sensing the AIEC metabolite propionate, which induces Th17 cells and drives intestinal inflammation (Viladomiu et al., 2021). Moreover, ETBF is strongly associated with the development of IBD, colitis associated cancer (CAC), and CRC. However, the mechanisms by which ETBF induces intestinal inflammation and tumorigenesis are unclear. *B. fragilis* contains the virulence gene *B. fragilis toxin (BFT)*, which encodes the virulence protein BFT, interacting with the membrane receptors of IECs and immune cells *via* the STAT3 and NF-κB pathways, causing the expression of IL-17 downstream, promoting the expression of inflammatory factors and thus influencing the development of CRC (Chung et al., 2018). Recent study has discovered that ETBF-stimulated cells deliver miR-149-3p *via* exosomes and promote Th17 cell differentiation. ETBF induced CRC by suppressing miR-149-3p and promoting PHD finger protein 5A (PHF5A)-mediated histone acetyltransferase *KAT2A* mRNA alternative splicing (Cao et al., 2021). *Campylobacter concisus* is involved in a wide range of inflammatory diseases, including IBD. The *zonula occludens toxin (Zot)* gene, discovered in *Asiatic cholera* and had a pathogenic effect, is found in 30% of *C. concisus* (Zhang et al., 2014). In a large study of Danish subjects, 962 people were infected with *C. concisus* and 1725 with *C. jejuni*. The risk of colitis was significantly higher in *C. concisus*-infected subjects than in controls, with a risk ratio of 32.4 (Nielsen et al., 2020). This also implies that *C. Concisus* likely hasten the onset of IBD. Further research has revealed that the enteropathogenic Zot virulence protein of *C. concisus* disrupts the intestinal epithelial barrier and induces the production of pro-inflammatory cytokines, particularly TNF-α, by intestinal epithelial and macrophage cells (Mahendran et al., 2016). *Fusobacterium varium* was found in up to 54.6% of 163 inflamed mucosal samples from 152 UC patients in Japan using real-time quantitative PCR (Tahara et al., 2015). A genome-wide analysis of an *F. varium* Fv113-g1 isolate from a UC patient’s intestine revealed numerous virulence factors, including 44 *Type 5 secretion systems (T5SS)* and 13 *FadA adhesion gene* homologs in mucosal inflammation (Sekizuka et al., 2017). Some gut microbiota (for example, pks + *E. coli*) produce colibactin, a chemically unstable small molecule genotoxin whose genotoxicity to host cells can increase the host’s risk of CRC. The effects of colibactin on other microorganisms in the gut have received little attention. A recent study discovered that colibactin could target surrounding bacteria containing prophages and activate prophages within the bacteria, causing phage replication and bacterial lysis. The induction of prophages by colibactin-producing bacteria has been observed in various human and gut-associated phage-bacterial systems and in mouse fecal flora. These findings shed light on the potential mechanisms by which colibactin influences gut microbiota and the relationship between bacterial products and phagosomal behavior. As a result, it is possible that bacteria evolved this genotoxin to inhibit other bacteria rather than poison the host and that the cancer-promoting effects of colibactin are a “misuse” of the host (Silpe et al., 2022). *Enterococci* are a group of bacteria widely found in the intestines of humans and other animals that cause multi-drug resistant infections. However, a family of potent protein-like toxins specifically targeting human and animal cells has not been previously identified in *Enterococcus* spp. Min’s group have recently identified and resolved a novel family of *Pore-forming toxins (Epxs)* expressed in *Enterococcus spp*. They discovered that Epx is a subclass of small β-barrel pore-forming toxins, with the β-barrel serving as the top structural domain and forming homo-octameric pores. The whole-genome CRISPR-Cas9 screen revealed that Epx2/3 binds to human leukocyte antigen class I (HLA-I). Through Epx2 virulence, Epx2+ *E. faecalis* strains harm peripheral blood mononuclear cells (PBMCs) and intestinal-like organs (Xiong et al., 2022). *Ruminococcus gnavus* is a type of commensal bacteria found in the human intestine. Several studies have found that they are significantly more abundant in IBD patients than in healthy subjects. *R. gnavus*. Can reduce intestinal mucin, which is mediated by intramolecular trans-sialidase (RgNanH; Tailford et al., 2015). Moreover, *R. gnavus*. Can stimulate DCs *via* the production of inflammatory polysaccharides, increasing the inflammatory cytokine TNF-α, promoting IBD’s development (Henke et al., 2019). Other bacteria that potentially contribute to IBD are *Helicobacter hepaticus* and *H. bilis*, both of which are from the genus *Helicobacter.* Bacteria from the genus *Helicobacter* that are not *H. pylori* often positively associated with IBD. In contrast, *H. pylori* and IBD show a negative correlation, meaning that people infected with H. *pylori* are less likely to develop IBD, possibly due to the ability of *H. pylori* to inhibit the growth of other bacteria. According to one study, in a mouse model of *IL-10* gene-deficient colitis, *H. hepaticus* induced inflammatory Th17, increased IL-17A and IFN-γ expression, and aided in the development of colitis (Xu et al., 2018). Some gut microbiota can colonize the intestinal mucus layer and feed on mucin, a major component of the mucus layer. Mucin is a heavily glycosylated protein with numerous O-glycan chains. Mucin O-glycan ends are frequently sulfated in the distal colon. Bacterial degradation of colonic mucin requires specific sulfatases to remove sulfate groups on O-glycans. However, little is known about the bacterial sulfate esterases that act on colonic mucin and how they work. A recently published study discovered a sulfate esterase expressed by *Bacillus thetaiotaomicron*, a common human intestinal commensal that uses sulfated colonic mucin O-glycans as its sole carbon source. The knockout of sulfate esterases from *B. thetaiotaomicron* revealed that one of the enzymes (BT1636^3S-Gal) is required to utilize sulfated mucin O-glycan and plays an important role in the intestinal colonization of the bacterium (Luis et al., 2021). These findings shed light on how gut microbiota degrade colonic mucins and the mechanisms associated with bacterial colonization and IBD. Blocking this enzymatic pathway could be a potential intervention strategy to prevent pro-IBD bacteria from disrupting the intestinal mucus layer barrier. *Fusobacterium nucleatum* infection may contribute to the worsening of UC, but its mechanism is not yet clear. Dong’s team recently published findings that EVs of *F. nucleatum* could promote the expression of pro-inflammatory factors (IL-1β, IL-6, TNF-α) and down-regulate the expression of anti-inflammatory factors (IL-10) and tight junction proteins (ZO-1 and occludin) in IECs by down-regulating the expression of *miR-574-5p* to promote the activation of autophagy, leading to barrier dysfunction and worsen DSS-induced colitis in mice (Wei et al., 2022). Furthermore, another recent study discovered that *Eggerthella lenta* could promote colitis in mice by activating Th17 cells and promoting IL-17a production through its expression of a drug-metabolizing enzyme, Cardiac glycoside reductase (*cgr2*). *E. lenta* was found to be enriched in patients with IBD, while the expression of the *cgr2* gene was increased in patients with rheumatoid arthritis. Cgr2 metabolizes steroidal saponins, which inhibit Th17 cell activity and are negatively associated with IBD severity. Furthermore, dietary arginine supplementation inhibited cgr2-induced Th17 cell activation and colitis in mice (Alexander et al., 2022).

## Targeting potential modulators and signaling pathways in intestinal epithelial cells to treat inflammatory bowel disease

Recent advances in various treatments have directly changed the clinical management model for IBD patients. The introduction of immunosuppressants and biologics significantly reduces the use of corticosteroids. In addition, the alpha 4β7 integrin blocker vedolizumab has also been included in the clinical IBD treatment. While these strategies expand the arsenal of IBD physicians, a significant percentage of patients do not respond to these treatments (Singh et al., 2018). Several new cytokine inhibitors, modulators of cytokine signaling pathways, inhibitors of transcription factors, and new anti-adhesion and anti-T cell activation and migration strategies are undergoing clinical trial evaluation. More therapeutic targets and signaling pathways need to be found to provide patients with more choices and personalized services in treatment decisions (Table 1).

**Table 1:** Targeting IEC modulators or signaling pathways to treat IBD.

Modulators/pathways	Mode of action	Biological process	References
Creatine transporter protein SLC6A8	Promote expression and localization of tight junction proteins Claudin1/2 and actin polymerization	Regulate energy balance and maintain barrier formation and healing function	Hall et al. (2020)
Golgi membrane protein 1 (GOLM1)	Regulate the balance of the Notch signaling pathway	Maintain barrier homeostasis	Pu et al. (2021)
Myosin light chain kinase splice variant (MLCK1)	TNF-induced MLCK1 recruitment to peri-junctional actomyosin ring (PAMR) mediates MLC phosphorylation.	Maintain barrier function	Graham et al. (2019)
	Interact with FKBP8 PPIase for MLCK1 recruitment and MLC phosphorylation		
miR-181 family members	Regulate Wnt and immune processes	Promote intestinal recovery after injury	Jimenez et al. (2022)
Histone H3K9 methyltransferase SETDB1	Silence endogenous retroviruses to inhibit DNA damage	Maintain IECs survival, barrier function, and homeostasis	Juznic et al. (2021)
m6A methyltransferase METTL14	Regulate *NF-kB* mRNA stability, affecting the activity of the NF-kB pathway and inhibiting TNF-mediated apoptosis	Regulate the development and apoptosis of colonic epithelial cells and colonic stem cells	Zhang et al. (2022)
DNA methyltransferase 3A (DNMT3A)	DNMT3A deficiency leads to hypomethylation	Maintain intestinal epithelial barrier function and regeneration	Fazio et al. (2022)
Special AT-rich sequence-binding protein 2 (SATB2)	Increase the expression of the Cl-/HCO3-transport protein SLC26A3	Maintain diversity and composition of the gut microbiota	Ni et al. (2021)
5-Hydroxytryptamine (5-HT)	Reduce TGF-β secretion and inhibit the TGF-β/SMAD pathway	Tumor-suppressive effects at the initiation stage of CAC	Mao et al. (2022)
	Enhance AKT activity through 5-HT2B and activate the IL-6/STAT3 pathway	Promote tumor progression in the later stages of CAC	
Spermidine oxidase (SMOX)	Promote spermidine production and inhibit alpha-defensin expression	Maintain diversity and composition of the gut microbiota	Gobert et al. (2022)
Stromal interaction molecule 1 (STIM1)	STIM1 deficiency controls the stimulation of the intestinal epithelium by reducing endoplasmic reticulum stress caused by an imbalance in Ca2+ homeostasis	Maintain intestinal barrier	Liang et al. (2022)
RhoB	Reduced or absent RhoB inhibits the Wnt pathway and activates the p38 MAPK pathway	Promote goblet cell differentiation and IECs proliferation, increase SCFA-producing bacteria abundance and elevate the levels of SCFAs and receptors	Yang et al. (2022)
Leukemia inhibitory factor (LIF)	Inhibit Th17 cells differentiation by phosphorylating and activating STAT4 and suppressing STAT3-induced IL17 gene expression	Alleviate colonic inflammation	Zhang et al. (2019)
Alpha B-crystallin (CRYAB)	Reduce cellular inflammatory factor (TNF-α, IL-6, IL-1β, IL-8) production by inhibiting IKKβ activity	Protect barrier integrity	Xu et al. (2019)
deSUMOylase SENP7	Ubiquitin ligase SIAH2 downregulates SENP7 through ubiquitination, which reduces pro-inflammatory mechanisms through γδ T cells.	Control intestinal inflammation	Suhail et al. (2019)
Epithelial NF-κB2 signaling	Amplify RelA activity and increase RelA-driven inflammatory gene expression	Induce abnormal intestinal inflammation	Chawla et al. (2021)
Guanylate cyclase C (GC-C)	Regulate intestinal ion and fluid secretion through cGMP production and activation of cGMP-dependent protein kinase II	Loss of overall internal environmental homeostasis, fluid ion imbalance, and dysregulation of the intestinal microbiota	Mishra et al. (2021)
Epithelial autonomous NAIP/NLRC4 inflammasomes	Drive IEC pyroptosis or apoptosis and promote IEC expulsion	Mitigate TNF-induced disruption of the intestinal epithelial barrier	Fattinger et al. (2021)
NLRP3 Inflammasomes	miR223 mediates NLRP3 regulation and N*LRP3* pathway blockage reduces IL-1β production	Inhibition of inflammasomes suppresses excessive inflammatory responses in the intestine	Kanneganti (2017)
ADP/P2Y1	ADP activates NLRP3 inflammasomes *via* P2Y1 receptors and promotes NLRP3 inflammasomes component ASC phosphorylation to increase IL-1β production		Zhang et al. (2020)
NLRP6 inflammasomes	Deubiquitinating enzyme Cyld binds to NLRP6, removes its ubiquitination modifications, inhibits NLRP6-ASC inflammasome formation, and regulates the maturation process of IL-18.		Mukherjee et al. (2020)
Nicotinamide adenine dinucleotide phosphate (NADPH) oxidase 1 (NOX1)	The absence of intestinal epithelial NOX1 combined with TNFα stimulation alters the stem cell microenvironment and stem cell differentiation	Promote lymphoplasmacytosis	Hsu et al. (2022)
Superoxide dismutase 1 (SOD1)	Restoring SOD activity inhibits p38-MAPK/NF-κB signal-mediated inflammation and apoptosis.	SOD1 deletion enhances oxidative stress and disrupts the intestinal epithelial barrier, reduces antioxidant enzyme activity, and increases colonic infiltration of pro-inflammatory immune cells	Hwang et al. (2020)
Multidrug resistance 1 (MDR1)	Reduce mROS	Protect mitochondria against xenotoxins through its efflux function	Ho et al. (2018)
3-mercaptopyruvate sulfurtransferase (MPST)	Regulate the AKT/apoptotic axis in IECs, inhibit pro-inflammatory cytokines expression, ROS production	Protect the intestine from inflammation and apoptosis incidence	Zhang et al. (2022)
Reductively modified albumin (r-Alb)	Inhibit cellular ROS and superoxide production through sulfhydryl (-SH)	Reduce the cellular damage caused by oxidative stress	Yang et al. (2021)

An essential function of the intestinal epithelium is to act as a barrier limiting the interaction between the luminal contents, such as intestinal microorganisms, and the immune system, absorbing nutrients while limiting microbial translocation (Allaire et al., 2018). Impaired intestinal barrier function leads to increased membrane permeability of IECs, which promotes the entry of intraperitoneal bacteria, toxins, and macromolecules into the body, thus inducing an inflammatory immune response. Intestinal epithelial barrier injury is of great interest in the study of the cellular and molecular pathogenesis of IBD, and reconstruction and regeneration of the intestinal epithelium is essential to restore intestinal homeostasis after injury (Sommer et al., 2021). Patients with IBD exhibit defects in multiple components of the mucosal barrier. Future research should focus on the modulation of the intestinal epithelial barrier as a possible therapeutic target for IBD.

By analyzing the function of the creatine transporter protein SLC6A8 (also known as CRT) in IECs, a recent study found that CRT is localized around tight junction proteins and can maintain intestinal epithelial barrier function by regulating the energy balance of IECs to promote the expression and localization of tight junction proteins. The deficiency of CRT inhibited creatine uptake, barrier formation, and healing function in IECs (Hall et al., 2020). Mechanistically, CRT deletion increased *Claudin-2* expression and decreased *Claudin-1* expression to promote the “leaky gut” phenotype, altered the localization of tight junction proteins, and inhibited actin polymerization. The study also found that the expression of *CRT* was significantly reduced in the colon tissue of IBD patients, suggesting that CRT may be a potential target for treating IBD (Hall et al., 2020). Recently, the protective role of IECs Golgi membrane protein 1 (GOLM1) in IBD has been demonstrated. IECs-specific deletion of GOML1 leads to greater susceptibility to DSS-induced colitis and Azoxymethane (AOM)/DSS-induced colonic carcinogenesis in mice. GOLM1 interacts with the Notch2 intracellular structural domain to maintain intestinal epithelial barrier homeostasis by regulating the balance of the Notch signaling pathway in IECs, thereby inhibiting the development of colitis and colorectal carcinogenesis (Pu et al., 2021). Moreover, myosin light chain kinase (MLCK) is also a key regulator of intestinal barrier function. A unique structural domain within the MLCK splice variant MLCK1 mediates its recruitment to the tightly linked peri-junctional actomyosin ring (PAMR). A small molecule divertin was identified previously through screening that blocked acute TNF-induced MLCK1 recruitment, downstream myosin light chain (MLC) phosphorylation, barrier dysfunction, and diarrhea *in vitro* and *in vivo*, and inhibited the progression of experimental IBD (Graham et al., 2019). Another recent study has found that MLCK1 can interact with the tacrolimus-binding protein FKBP8 peptidyl-prolyl cis/trans isomerase (PPIase) structural domain and that these interactions are critical for MLCK1 recruitment, MLC phosphorylation, and TNF-induced barrier loss. Tissue biopsies from CD patients revealed an increased number of intercellularly linked MLCK1-FKBP8 interactions compared to controls. Blocking MLCK1-FKBP8 binding reversed MLCK1-FKBP8 interactions, MLCK1 recruitment, and barrier loss *in vitro* and *in vivo* (Zuo et al., 2022). These studies provide a new potential therapeutic target for diseases related to intestinal barrier dysfunction, such as IBD.

There are fewer studies on post-transcriptional regulation in the maintenance of intestinal homeostasis and the development of colitis. A recently published study showed that the conserved miR-181 family members in the intestinal epithelium are down-regulated in IBD and mouse colitis samples, and their expression promotes intestinal recovery after injury by regulating Wnt and immune processes, which could be a potential new target for the treatment of intestinal inflammation (Jimenez et al., 2022). Furthermore, SETDB1 is a histone H3K9 methyltransferase that plays a regulatory role in intestinal epithelial homeostasis and IBD. Recent studies reveal that SETDB1 can maintain intestinal epithelial homeostasis by silencing endogenous retroviruses to inhibit DNA damage. Mice specifically deficient in SETDB1 in the intestinal epithelium exhibited impaired intestinal epithelial differentiation, impaired intestinal barrier, enhanced intestinal inflammation, and reduced survival. Meanwhile, some missense mutations associated with SETDB1 loss of function were significantly enriched in IBD patients (Juznic et al., 2021). Taken together, the results of this study suggest that SETDB1 may be a potential target for the treatment of IBD. In recent years, many studies have found that the methylation modification of RNA N6-methyladenosine (m6A) is involved in the maintenance of many types of stem cells, but whether RNA m6A also plays a vital role in intestinal stem cells is unclear. Recently, Li’s group found that m6A methyltransferase METTL14 plays an essential role in colonic stem cell self-renewal, and it may regulate the development and apoptosis of colonic epithelial cells and colonic stem cells by regulating the stability of *NF-kB* mRNA, affecting the activity of NF-kB pathway, and inhibiting TNF-mediated apoptosis (Zhang et al., 2022). Therefore, m6A is closely related to the development of colitis and can be used as a potential clinical target for the treatment of colitis. DNA methyltransferase 3A (DNMT3A) is involved in DNA methylation modifications and its genetic variants are associated with IBD. A recent study observed downregulation of *DNMT3A* expression in IECs in CD patients. Mechanistically, DNMT3A deficiency leads to hypomethylation, which impairs the function and regeneration of the intestinal epithelial barrier, making mice more susceptible to DSS-induced colitis, implying a role for impaired epithelial DNMT3A function in the etiology of IBD (Fazio et al., 2022).

Recently, the role of some regulatory factors known to be associated with colorectal carcinogenesis and development in IBD has also been revealed. Special AT-rich sequence-binding protein 2 (SATB2) is a potential diagnostic and prognostic marker for CRC, but its role in colitis and CAC is unclear. A recent study found that intestinal epithelial cell-specific SATB2 deficiency worsened colitis and CAC in mice by altering the diversity and composition of the gut microbiota and decreasing the expression of the Cl-/HCO3-transport protein SLC26A3 (Ni et al., 2021). Moreover, 5-hydroxytryptamine (5-HT) is commonly associated with CAC, and the conclusions on how 5-HT affects CAC are not yet uniform. 5-HT2B is one of the receptors for 5-HT, which is expressed in IECs. A recent study revealed that 5-HT/5-HT2B/TGF-β signaling exerts tumor-suppressive effects at the initiation stage of CAC while promoting tumor progression in the later stages. 5-HT2B-deficient IECs, on the one hand, reduce TGF-β secretion and inhibit the TGF-β/SMAD pathway; on the other hand, it enhances AKT activity through 5-HT2B and thus activate the IL-6/STAT3 pathway, enhance the intestinal inflammatory response, and exacerbates IEC destruction, thus promoting CAC tumorigenesis. Antibody blockade of IL-6 reverses the promoting effect of 5-HT2B deficiency on CAC development. TCGA data show a positive correlation between 5-HT2B expression and prognosis in CRC patients (Mao et al., 2022). Previous studies have shown that spermidine oxidase (SMOX) promotes spermidine production and regulates colitis. In a recent study, SMOX deficiency in mice worsened DSS-induced colitis and AOM/DSS-induced colonic tumorigenesis, increased alpha-defensin expression, and induced dysbiosis of gut microbiota, leading to reduced abundance of *Prevotella* and increased *Proteobacteria* and *Deferribacteres.* In contrast, spermidine supplementation reversed the above phenotype. In addition, down-regulation of *SMOX* expression was observed in inflamed colonic tissues from patients with UC and patients with colitis-associated heterotypic hyperplasia (Gobert et al., 2022). Furthermore, stromal interaction molecule 1 (STIM1) is an integral component of the store-operated calcium entry (SOCE) process that promotes CRC and T-cell-mediated inflammatory diseases. However, it is unclear whether STIM1 in IECs is involved in the pathological process of IBD. Recent studies have found that STIM1 deficiency in the intestinal epithelium in colitis controls the stimulation of the intestinal epithelium by commensal bacteria by reducing endoplasmic reticulum stress caused by an imbalance in Ca2+ homeostasis, reducing loss of goblet cells, and maintaining the integrity of the mucus layer (Liang et al., 2022). STIM1 is a vital regulator of maintaining the intestinal barrier and a potential target for IBD treatment. RhoB, a member of the small Rho GTPase family, exhibits rapid upregulation when the organism is induced by genotoxic stress, LPS, inflammatory cytokines, growth factors and toxins, and is involved in a variety of cellular processes; however, the role of RhoB in colitis remains unclear. Recently, Wang Quan’s team found that RhoB is significantly upregulated in UC, and that reduced or absent RhoB promotes goblet cell differentiation and proliferation of IECs by inhibiting the Wnt pathway and activating the p38 MAPK pathway. In addition, decreased RhoB increased the abundance of SCFA-producing bacteria and elevated the levels of SCFAs and their receptors. These findings suggest that RhoB is a potential molecular marker for the diagnosis of IBD, as well as a potential therapeutic target (Yang et al., 2022).

The intestine is the fastest self-renewing tissue in the adult mammalian organism, and its primary stem cells are located in the crypt of the intestinal villi. Stem cells in this location have a high capacity for self-renewal and differentiation and can differentiate into intestinal epithelial absorptive cells and intestinal epithelial endocrine cells after 4–5 mitotic divisions. Among them, Paneth cells and Tuff cells are involved in the maintenance of intestinal stem cells. Different subtypes of cells differentiated from intestinal stem cells are essential in maintaining intestinal homeostasis by producing a mucus layer covering the surface of the intestinal epithelial cells, recognizing pathogens, and producing antimicrobial peptides to ensure an effective immune response (Peterson and Artis, 2014). Moreover, a recent study found that leukemia inhibitory factor (LIF), a cytokine in the IL-6 family secreted by IECs, alleviates colonic inflammation by inhibiting the differentiation of Th17 cells in a mouse model of colitis. Mechanistically, LIF inhibits the differentiation of Th17 cells by phosphorylating and activating STAT4 and suppressing STAT3-induced *IL17* gene expression (Zhang et al., 2019). The results of this study provide new potential therapeutic targets for the treatment of IBD. Alpha B-crystallin (CRYAB) is a small heat shock protein that plays a protective role in intestinal inflammation. A recent study found that *CRYAB* expression was significantly reduced in both IBD patients and DSS-induced colitis mouse models and negatively correlated with TNF-α and IL-6 levels; CRYAB could reduce cellular inflammatory factors (TNF-α, IL-6, IL-1β, IL-8) production *in vitro* by inhibiting IKKβ activity, and in colitis mice *in vivo* (Xu et al., 2019). This suggests that CRYAB may be a potential therapeutic target for IBD, as it protects the integrity of the intestinal barrier and alleviates colitis symptoms. Previous studies have found that epithelial deSUMOylation is associated with IBD. The deSUMOylase SENP7 and its interacting group regulate epithelial-immune cross-reactivity. In healthy cells, the ubiquitin ligase SIAH2 negatively regulates SENP7 through ubiquitination. Upregulation of epithelial SENP7 induces pro-inflammatory mechanisms through γδ T cells. Knockdown of SENP7 or clearance of γδ T cells suppressed DSS-induced intestinal inflammation; upregulation of *SENP7* expression was strongly statistically correlated with higher clinical disease indices in IBD patients (Suhail et al., 2019). This study shows that epithelial SENP7 is necessary to control intestinal inflammation and highlights its importance as a potential drug target.

In addition, excessive activation of RelA/NF-κB causes abnormal inflammation associated with IBD. The canonical NF-κB module regulates intranuclear activation of RelA dimers and induces e pro-inflammatory gene expression, and uncontrolled RelA activity induces abnormal intestinal inflammation. A recently published study found that atypical NF-κB2 signaling amplifies RelA activity in colonic epithelial cells, increases RelA-driven inflammatory gene expression, and exacerbates intestinal pathogenesis in IBD patients and mice with colitis, suggesting that the atypical NF-κB2 pathway may be a potential therapeutic target for inflammatory diseases (Chawla et al., 2021). Activating mutations in guanylate cyclase C (GC-C), a target receptor for the gastrointestinal peptide hormone guanylate and urinary guanylate, and bacterial heat-stable enterotoxins can both cause early-onset diarrhea and chronic IBD. GC-C regulates intestinal ion and fluid secretion through cGMP production and activation of cGMP-dependent protein kinase II. A recent study constructing activating mutant mice with the *guanylate cyclase C (Gucy2c)* gene found that mutant mice have increased intestinal cGMP, leading to loss of overall internal environmental homeostasis, fluid ion imbalance, dysregulation of the intestinal microbiota, and susceptibility to colitis, suggesting that gut-associated cGMP signaling pathway may mediate colitis and flora dysbiosis (Mishra et al., 2021).

Inflammasomes are an important natural immune component expressed in both immune cells and non-immune cells (Lamkanfi and Dixit, 2014). By regulating the degree of inflammation and cell death in response to pathogen-associated molecular patterns (PAMPs) or danger-associated molecular patterns (DAMPs), they can either protect or harm the host (Man, 2018). Inflammasomes play an important role in inflammation and pathogen clearance. However, the mechanisms by which endogenous danger signals activate inflammasomes and the association of inflammasomes with inflammatory diseases have not been clarified. Earlier studies have shown that *Salmonella typhimurium* expresses a specific PAMP, a ligand for NLR family of apoptosis inhibitory proteins (NAIPs), during infection of IECs and high expression of NAIP/NLRC4 inflammasomes in IECs specifically inhibits the dissemination of *S. typhimurium* from the intestine to the whole body (Rauch et al., 2017; Hausmann et al., 2020). A recent study demonstrated that epithelial autonomous NAIP/NLRC4 inflammasomes mitigate TNF-induced disruption of the intestinal epithelial barrier by driving IEC pyroptosis or apoptosis and promoting IEC expulsion (Fattinger et al., 2021). *miR-223* expression is increased in inflammatory tissues in IBD (Zhang et al., 2021). NLRP3 is a component of inflammasomes, and *NLRP3* expression is elevated in colon and myeloid cells in miR-223-deficient environments. Drugs block the N*LRP3* pathway, reduce IL-1β production, and attenuate colitis (Kanneganti, 2017). Recently, Zhang et al. revealed the mechanism by which endogenous danger signals activate inflammasomes to promote IBD. ADP, a danger signal released during colonic injury from IECs, activates NLRP3 inflammasomes *via* P2Y1 receptors and promotes phosphorylation of NLRP3 inflammasomes component ASC to increase IL-1β production, thereby worsening DSS-induced colitis in mice. Extracellular ADP activates NLRP3 inflammasomes *via* P2Y1 receptor-mediated calcium signaling, and deletion of P2Y1 receptors or treatment with P2Y1 receptor inhibitors inhibits NLRP3 inflammasomes activation to alleviate colitis (Zhang et al., 2020). The results of this study suggest that ADP/P2Y1 may serve as a potential therapeutic target for IBD. NLRP6 inflammasomes regulate the intestinal inflammatory response and the organism’s resistance to microbial responses, but the molecular mechanism of how to inhibit NLRP6 function to avoid tissue damage caused by excessive inflammatory response is not clear. A recent study showed that the deubiquitinating enzyme Cyld binds to NLRP6, removes its ubiquitination modifications, inhibits NLRP6-ASC inflammasomes formation, regulates the maturation process of IL-18, and suppresses excessive inflammatory responses in the intestine (Mukherjee et al., 2020). This finding may provide new ideas for the treatment of intestinal inflammation.

The intestinal epithelium is constantly exposed to inducers of ROS, such as commensal microorganisms (Kajino-Sakamoto et al., 2010). In addition to an excessive inflammatory response, oxidative stress is considered a major feature of IBD (Tian et al., 2017). In the gastrointestinal tract, ROS production by nicotinamide adenine dinucleotide phosphate (NADPH) oxidase (NOX/DUOX) is a key biological mechanism regulating pathogen killing, host–microbe interactions and tissue repair after injury (Aviello et al., 2019). ROS production *via* enterocyte NOX1 has been shown to transduce microbial signals that promote epithelial wound healing (Alam et al., 2014) and mutations in genes that induce ROS production, such as Nox1, are highly associated with IBD (Aviello et al., 2019; Hsu et al., 2022). Mechanistically, the absence of intestinal epithelial NOX1 combined with TNFα stimulation can alter the stem cell microenvironment and stem cell differentiation, promote the number of lymphoplasmacytes and thus accelerate the progression of colitis, which may provide new insights into the prevention and treatment of IBD (Hsu et al., 2022). Superoxide dismutase 1 (SOD1) is one of the three superoxide dismutases responsible for the destruction of free superoxide radicals in the body. However, the role of SOD1 in oxidative stress in colitis is unclear. Hwang et al. found that SOD1 deletion enhances oxidative stress in mice and disrupts the intestinal epithelial barrier, reduces antioxidant enzyme activity, and increases colonic infiltration of pro-inflammatory immune cells to worsen DSS-induced colitis in mice. Restoring SOD activity can inhibit p38-MAPK/NF-κB signal-mediated inflammation and apoptosis, thereby alleviating colitis (Hwang et al., 2020). In mice, loss of multidrug resistance 1 (MDR1) function leads to colitis similar to human IBD. Ho et al. showed that MDR1 has a protective effect on mitochondria, where MDR1 deficiency leads to mitochondrial dysfunction, while increased mROS drive the development of colitis. Since MDR1 protects against xenotoxins primarily through its efflux function, the results of this study suggest a unique mitochondrial toxin genetic susceptibility interaction leading to mitochondrial dysfunction, a novel pathogenic mechanism that could provide many new therapeutic opportunities for IBD (Ho et al., 2018). Moreover, endogenous hydrogen sulfide (H2S) has anti-inflammatory activity in IBD, and the role of 3-mercaptopyruvate sulfurtransferase (MPST), a key enzyme regulating endogenous H2S biosynthesis, in IBD is not yet clear. A recent study found that MPST likely protects the intestine from inflammation by regulating the AKT/apoptotic axis in IECs, inhibiting the expression of pro-inflammatory cytokines, ROS production, and the incidence of apoptosis, providing a new therapeutic strategy for colitis (Zhang et al., 2022). Albumin is the most abundant matrix protein in the body and performs a variety of biological functions, including the ability to resist oxidative stress through free sulfhydryl groups (-SH). Oxidative stress is an important feature of colitis, and studies have shown that colitis can significantly reduce albumin and increase oxidized albumin (Krzystek-Korpacka et al., 2008; Khan et al., 2017). However, no studies have yet explored the potential efficacy of albumin in the treatment of colitis. A recent work showed that DSS-induced administration of reductively modified albumin (r-Alb) to mice with colitis effectively improved the colitis phenotype by the mechanism that r-Alb inhibits cellular ROS and superoxide production through sulfhydryl (-SH) and reduces the cellular damage caused by oxidative stress. The results of this study provide a new intervention technique for the clinical treatment of colitis, while r-Alb has a promising application as an effective antioxidant (Yang et al., 2021).

## Conclusion

Multiple factors are involved in the pathogenesis of IBD, such as genetic, environmental, infectious, and immunological factors, among which intestinal inflammation and immune dysfunction play an important role. In IBD patients, molecules such as SCFAs, bile acids and microbial tryptophan metabolites are altered (Sun et al., 2017; Heinken et al., 2019; Scott et al., 2020). These microbe-derived compounds function as signaling molecules, mediating host-microbiota communication and regulating immune homeostasis. Recent research suggests that SCFAs, particularly butyrate, have immunomodulatory properties. Furthermore, SCFAs can activate the signaling cascade that controls immune function *via* GPCRs. With the advancement of molecular biology techniques, the value of epigenetics as one of the pathways regulating gene expression in IBD has been gradually explored and established to be particularly relevant to IBD. Butyrate has recently been discovered to regulate epithelial gene expression or immune cells *via* epigenetic pathways. Besides, the latest evidence suggests that specific bile acid metabolites, tryptophan metabolites, and PSA interact with adaptive immune cells to improve the inflammatory environment of IBD. In addition to traditional bacterial metabolites, certain new bacteria species and their effector molecules have been identified to regulate immune factors release and improve IBD by binding to IECs or immune cell surface receptors. On the contrary, many new bacteria species and the virulence factors they contain potentially promote IBD by disrupting epithelial barrier, promoting pro-inflammatory factors secretion, and interfering normal gut microbiota. Recent research has confirmed the importance of miRNAs in targeting specific molecules in signaling pathways that regulate intestinal barrier homeostasis, inflammation, and autophagy. Several studies have found specific miRNAs linked to IBD and attempted to use them as diagnostic biomarkers (Soroosh et al., 2018).

The long-term persistence of chronic inflammation in IBD is a major contributor to tumor transformation and the development of CAC. In the past decades, significant progress has been made in the immunological mechanisms of IBD, providing new strategies and ideas for treating IBD. The subsequent introduction of biologics, such as TNF-α blocker, replaced nonselective anti-inflammatory corticosteroids in IBD management. However, these therapies still have the potential for primary unresponsiveness, secondary loss of response, opportunistic infections, and cancer development. Therefore, there is an urgent need to develop novel and effective therapies that target specific signaling pathways in the pathogenesis of IBD. Disruption of the intestinal barrier, abnormal death of epithelial cells, and subsequent inflammation are at the heart of chronic inflammatory and infectious gastrointestinal diseases (Patankar and Becker, 2020). Recent studies have shown that the Notch, Wnt, and NfkB signaling pathways are involved in the pathogenesis of IBD and different upstream signaling molecules can activate these pathways, thus serving as potential targets for the treatment of IBD. Moreover, RNA methylation is a new class of RNA epigenetic modifications discovered in recent years and can regulate mRNA expression. One of the most common modifications is m6A. There is a growing body of research involving RNA methylation and disease, covering tumors (Su et al., 2019), neurological disorders (Jiang et al., 2022), obesity (Li et al., 2020), and immune response (Wang et al., 2020), but the relationship with IBD needs further investigation. Elevated levels of NLRP3 inflammasome and pro-inflammatory cytokines are the main pathological mechanisms of IBD (Zhen and Zhang, 2019). Therefore, targeting NLRP3 inflammasome offers a promising strategy for the treatment of IBD. Chronic intestinal inflammation is related with the overproduction of ROS, which leads to oxidative stress. Recent studies have identified several key molecules that play varying degrees of regulatory functions in the production of ROS and could be explored as possible targets for IBD therapy. In the future, altering specific genetic loci may be a promising therapeutic approach for IBD. In addition, novel antibodies or inhibitors, combination therapy regimens, and multifactor blockers are also expected to break the bottleneck of IBD treatment and improve disease for patients with IBD. Furthermore, in the past three decades, there has been a rapid development in the clinical diagnosis and treatment of IBD. However, numerous issues still need to be explored in depth, such as genetic testing, serological markers in the disease population, imaging of the disease, endoscopic assessment, monitoring, drug specificity, and FMT. It is the future trend to further develop relatively specific novel drugs, develop IBD assessment models through extensive sample population studies, and provide personalized treatment to patients.

## Author contributions

BL and CnW wrote the paper. CoW, WS, and XH made figures. WC made the table. NL and DX conceived of the presented idea. All authors contributed to the article and approved the submitted version.

## Funding

This work is financially supported by the Qingdao Postdoctoral Applied Research Project (grant no. RZ2200001423).

## Conflict of interest

The authors declare that the research was conducted in the absence of any commercial or financial relationships that could be construed as a potential conflict of interest.

## Publisher’s note

All claims expressed in this article are solely those of the authors and do not necessarily represent those of their affiliated organizations, or those of the publisher, the editors and the reviewers. Any product that may be evaluated in this article, or claim that may be made by its manufacturer, is not guaranteed or endorsed by the publisher.
